# Evaluation of the Acceptability of a Proposed, Instagram-Based, Randomized Controlled Trial for People With Asthma: Survey Study

**DOI:** 10.2196/24005

**Published:** 2021-09-30

**Authors:** Kerry A Spitzer, Brent Heineman, Marcella Jewell, Michael Moran, Peter K Lindenauer

**Affiliations:** 1 Institute for Healthcare Delivery and Population Science University of Massachusetts Medical School-Baystate Springfield, MA United States; 2 University of Connecticut School of Medicine Farmington, CT United States; 3 University of Massachusetts Medical School Worcester, MA United States; 4 Department of Medicine University of Massachusetts Medical School-Baystate Springfield, MA United States; 5 Department of Population and Quantitative Health Sciences University of Massachusetts Medical School Worcester, MA United States

**Keywords:** asthma, social media, Instagram, social support, digital storytelling, young adult

## Abstract

**Background:**

Asthma is a chronic lung disease that affects nearly 25 million individuals in the United States. More research is needed into the potential for health care providers to leverage existing social media platforms to improve healthy behaviors and support individuals living with chronic health conditions.

**Objective:**

In this study, we assessed the willingness of Instagram users with poorly controlled asthma to participate in a pilot randomized controlled trial that will use Instagram as a means of providing social and informational support. In addition, we explored the potential for adapting the principles of photovoice and digital storytelling to Instagram.

**Methods:**

We conducted a survey study of Instagram users aged 18-40 years with poorly controlled asthma in the United States.

**Results:**

Over 3 weeks of recruitment, 457 individuals completed the presurvey screener; 347 (75.9%) were excluded and 110 (24.1%) were eligible and agreed to participate in the study. Of the 110 individuals, 82 (74.5%) completed the study survey. The mean age of the respondents was 21 (SD 5.3) years. Among respondents, 56% (46/82) were female, 65% (53/82) were non-Hispanic White, and 72% (59/82) had at least some college education. The majority of respondents (67/82, 82%) indicated that they would be willing to participate in the proposed study.

**Conclusions:**

Among young adult Instagram users with asthma, there is substantial interest in participating in a pilot randomized controlled trial that will use Instagram to connect participants with peers and a health coach to share information about self-management of asthma and build social connection.

## Introduction

Asthma is a chronic lung disease that affects nearly 25 million individuals in the United States. The incidence of asthma varies across the United States, both geographically and socially; it is the highest in the Northeast and among vulnerable populations. For example, the incidence of asthma is higher among Puerto Ricans (14.2%) and African Americans (9.6%) than among non-Hispanic White adults (8.2%) [1]. Asthma can be a significant burden on both the individual and the health care system and leads to missed school and work and costly hospital visits [1]. Self-management is essential to controlling asthma and reducing interruptions to daily life. There is evidence that social support can affect self-management of asthma. For example, friends or family members may reinforce positive behaviors such as remembering to take medication. Similarly, peers may have a negative influence if they promote behaviors that go against health care providers’ recommendations [2]. How interaction with peers through social media could be harnessed to improve self-management is not well understood [3]. An increasing number of American adults are sharing health information on social media apps [4]. A study from Pew [4] found that 59% of adults in the United States have searched online for health information in the past year and 16% of those adults have tried to find others who might have the same health concerns. Young adults are more likely to use social media apps than the average adult. For example, while 35% of adults in the United States use Instagram, 74% of those aged 18-24 years use the photo-sharing app [5].

Some health care researchers have recognized social media to be a source of health information and a means of connection and have adapted the delivery of traditional behavior support interventions to social media or custom websites and apps [6-10]. The technology that has enabled the rise of social media—cameras in everyone’s pockets—has also removed barriers to the use of photovoice and digital storytelling, the community-based participatory action research methodologies that have traditionally relied on the use of film or digital cameras. Photovoice has been adapted to address diabetes and smoking cessation among young adults using Instagram and Facebook, but to our knowledge social media has not been harnessed to support individuals with asthma [11,12]. More research is needed into the potential for health care providers to leverage existing platforms to improve healthy behaviors and support individuals living with chronic health conditions such as asthma. In this study, we assessed the willingness of Instagram users with poorly controlled asthma to participate in a pilot randomized controlled trial that will use Instagram as a means of providing social and informational support. In addition, we explored the potential for adapting the principles of photovoice and digital storytelling to Instagram.

## Methods

### Recruitment

We conducted a web-based survey of young adults with poorly controlled asthma. To recruit Instagram users, we used Facebook Ads Manager, a Facebook-based platform that allows for the customization of targeted advertisements that run on Instagram. To enroll suitable participants, achieve our target sample size, and adhere to Facebook’s requirements, we requested that the study advertisement target individuals aged 18-40 years from the New England area with search results indicating interest in asthma and allergy friendly resources, Instagram, or health and wellness resources. We ran the advertisement on Instagram and Facebook from January 6, 2020, through January 26, 2020. We offered users the chance to win a $10 Amazon gift card on the completion of a brief survey about asthma and Instagram (Multimedia Appendix 1). Users who clicked on the advertisement were directed to a screening questionnaire on REDCap. Respondents were potentially eligible if they reported an active asthma diagnosis. We limited the study to individuals with poorly controlled asthma as measured by the Asthma Control Test in the screening questionnaire. A score of 19 or less indicates poorly controlled asthma. In addition, we only included individuals who lived in the United States and were willing to complete a brief questionnaire in English.

### Survey

The self-administered questionnaire was designed to take 10 minutes to complete and to gauge interest in a proposed study that will use Instagram to provide social and informational support to individuals with poorly controlled asthma. We described the study as follows:

We are planning a study to determine whether people with asthma benefit from sharing and reflecting on their condition using social media tools like Instagram. As part of this intervention, we will ask participants to follow a health coach from the study team on Instagram. The coach will post regularly about living with asthma, including strategies to achieve better control of symptoms. These posts will prompt participants to discuss living with asthma with other participants. The study will last 3 months. At the end of each month, participants will be asked to complete a self-reflection exercise about their asthma and experiences in the study. Participants will be compensated $75 for completing the study.

We then asked respondents a series of questions about the proposed study and asked them to respond with “Very willing,” “Somewhat willing,” “Somewhat unwilling,” or “Very unwilling.” We asked questions about the acceptability of the intervention, such as “As part of the study, how willing would you be to join an Instagram group, moderated by a health coach, with the goal of helping participants better control their asthma?” and “How willing would you be to post about your asthma on an account created for the purposes of the study?”

To elicit feedback on the proposed study, we asked both open- and close-ended questions. We asked, “What are your concerns about participating in the study described above, if any?” and allowed respondents to select multiple responses, including “Other” with the ability to write a response. The close-ended responses were “I don’t want to share photos relating to my asthma with others,” “I don’t think the compensation is enough,” “I don’t use Instagram enough,” “I don’t think this study will help me,” “I don’t have the time,” “I need more information,” and “No Concerns.” In addition, we asked the following open-ended questions: “Please comment on what (if any) elements of the proposed study interest you” and “Please use the provided space to share any additional thoughts you may have about how to make the proposed study better.”

We also assessed the severity of respondents’ asthma symptoms through the questions “In the last year, have you visited the emergency department for the purpose of treating your asthma?” and “In the last year, have you been hospitalized for your asthma?” In addition, 7 questions assessed the frequency and nature of Instagram use. We assessed self-efficacy for managing symptoms of chronic conditions using the 4-item Patient-Reported Outcomes Measurement Information System short form. We collected education, gender identity, ethnicity, race, and age demographics (Multimedia Appendix 2).

The Baystate Health Institutional Review Board reviewed this study and determined that it met the federal criteria for exemption. All procedures in this study were performed in accordance with the ethical standards of Baystate Health and the 1964 Helsinki declaration and its later amendments or comparable ethical standards. Informed consent was obtained from all participants included in the study.

### Statistical Analysis

We used descriptive statistics to summarize the study sample and determine the proportion of respondents willing to participate in the proposed study. Data management and quantitative analyses were conducted using Stata (Statistical Software: Release 16, StataCorp LLC). KAS conducted all statistical analyses. To summarize the open-ended responses, we used an inductive qualitative approach to generate themes from comments about interests and concerns related to the proposed study. Given the small number of open-ended comments about interests and concerns related to participating in the proposed pilot, a team of three research assistants and the clinical research coordinator reviewed all responses and agreed on emergent themes.

## Results

### Participant Demographics

**Figure 1:**
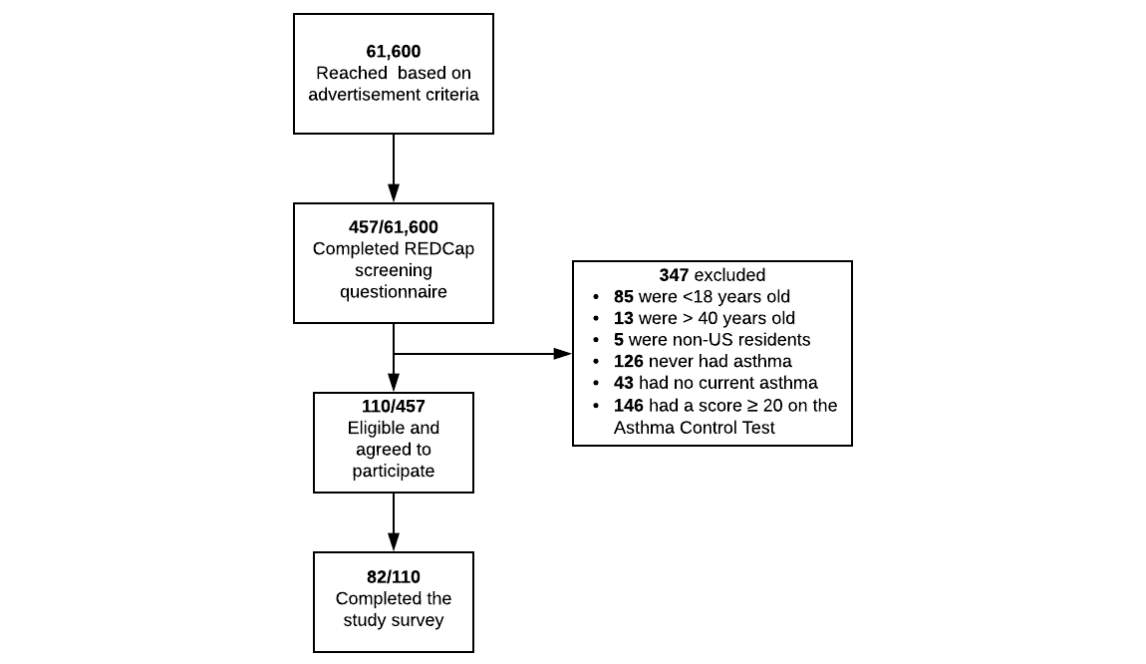
Study recruitment flow diagram.

Given our specifications and budget, our advertisements potentially reached 61,600 Facebook and Instagram users. Over 3 weeks of recruitment, 457/61,600 (0.74%) individuals completed the presurvey screener; 347 (75.9%) were excluded because of age under 18 years (85/347, 24.4%), age over 40 years (13/347, 3.7%), non-US resident status (5/347, 1.4%), no history of asthma (126/347, 36.3%), no active asthma diagnosis at the time of the survey (43/347, 12.3%), and well-controlled asthma (score ≥20 on the Asthma Control Test; 146/347, 42%). Of the 457 individuals, 110 (24.1%) were eligible and agreed to participate in the study; 82 of the 110 individuals (74.5%) completed the study survey (Figure 1).

The mean age of the respondents was 21 (SD 5.3) years. Among respondents, 56% (46/82) were female, 65% (53/82) were non-Hispanic White, and 72% (59/82) had at least some college education (Table 1). The majority of respondents (67/82, 82%) indicated that they were willing to participate in the proposed study. There were no significant differences between those willing and those unwilling to participate with regard to emergency department visit and hospitalization rates, Asthma Control Test score, frequency and nature of Instagram use, education, gender identity, ethnicity, race, age, and self-efficacy for managing symptoms.

Both groups reported similar rates of hospitalization and emergency department visits for asthma-related problems. Just under one-third of all respondents (26/82, 32%) had visited the ED, and 11% (9/82) had been hospitalized for asthma within the past year.

**Table 1:** Respondent characteristics.

Characteristics		Total (N=82), n (%)	Willing to participate (N=67), n (%)
**Highest degree or level of school completed**			
	Less than high school	6 (7)	4 (6)
	High school degree	14 (17)	13 (19)
	Some college but no degree	28 (34)	22 (33)
	Associate degree	5 (6)	4 (6)
	Bachelor degree	18 (22)	14 (21)
	Graduate degree	8 (10)	7 (10)
	Unknown	3 (4)	3 (4)
**Gender**			
	Male	23 (28)	19 (28)
	Female	46 (56)	37 (55)
	Transgender	6 (7)	5 (7)
	Nonbinary	4 (5)	3 (4)
	Unknown	3 (4)	3 (4)
**Race**			
	American Indian or Alaskan Native	2 (2)	2 (3)
	Asian	8 (10)	5 (7)
	Black or African American	7 (9)	6 (9)
	White	58 (71)	48 (72)
	Other	2 (2)	2 (3)
	Unknown	5 (6)	4 (6)
**Ethnicity**			
	Non-Hispanic/non-Latino	67 (82)	54 (81)
	Hispanic/Latino	11 (13)	10 (15)
	Unknown	4 (5)	3 (4)
**Age group (years)**			
	18-24	63 (77)	49 (73)
	25-29	5 (6)	4 (6)
	30-40	14 (17)	14 (21)

### Instagram Use

The majority of respondents (46/82, 56%) had private Instagram accounts. Few respondents (7/82, 9%) followed hashtags or accounts specifically related to asthma, but half of the respondents (41/82, 50%) followed more general hashtags related to health and wellness. Many of the survey respondents (67/82, 82%) reported that they use Instagram multiple times a day. Almost all respondents (79/82, 96%) indicated that they post on Instagram, though at varying frequencies; most (45/82, 55%) responded that they post monthly. Respondents also comment on other individuals’ posts (65/82, 79%). Those who indicated willingness to participate in the proposed study (67/82, 82%) reported Instagram use characteristics that were similar to those of the total respondent group (Table 2).

**Table 2:** Instagram use and willingness to participate in the proposed study.

Survey question and response		Total (N=82), n (%)	Willing to participate^a^ (N=67), n (%)
**How frequently do you open the Instagram app/log-onto Instagram?**			
	Multiple times a day	67 (82)	55 (82)
	About once a day	12 (15)	11 (16)
	A few times per week	2 (2)	1 (1)
	A few times per month	1 (1)	0 (0)
**Do you post on Instagram?**			
	No	3 (4)	2 (3)
	Yes	79 (96)	65 (97)
**If yes, how frequently?**			
	Daily	5 (6)	4 (6)
	Weekly	21 (26)	17 (25)
	Monthly	45 (55)	38 (57)
	Yearly	8 (10)	6 (9)
	N/A^b^	3 (4)	2 (3)
**Do you comment on posts?**			
	No	17 (21)	14 (21)
	Yes	65 (79)	53 (79)
**If yes, how frequently?**			
	Daily	15 (18)	13 (19)
	Weekly	31 (38)	28 (42)
	Monthly	19 (23)	12 (18)
	N/A	17 (21)	14 (21)
**Do you have a public or private Instagram account?**			
	Public	36 (44)	29 (43)
	Private	46 (56)	38 (57)
**Do you follow any hashtags or accounts related to asthma?**			
	No	75 (91)	61 (91)
	Yes	7 (9)	6 (9)
**Do you follow any hashtags related to health and wellness?**			
	No	41 (50)	31 (46)
	Yes	41 (50)	36 (54)
**As part of the study, how willing would you be to join an Instagram group, moderated by a health coach, with the goal of helping participants better control their asthma?**			
	Very willing	30 (37)	30 (45)
	Somewhat willing	36 (44)	32 (48)
	Somewhat unwilling	8 (10)	2 (3)
	Very unwilling	8 (10)	3 (4)
**How willing would you be to post about your asthma on an account created for the purposes of the study?**			
	Very willing	22 (27)	21 (31)
	Somewhat willing	31 (38)	28 (42)
	Somewhat unwilling	15 (18)	10 (15)
	Very unwilling	14 (17)	8 (12)
**How willing would you be to like, comment, or interact with the posts of others with asthma on a weekly basis?**			
	Very willing	37 (45)	35 (52)
	Somewhat willing	32 (39)	26 (39)
	Somewhat unwilling	7 (9)	3 (4)
	Very unwilling	6 (7)	3 (4)

^a^If a respondent answered “Very willing” or “Somewhat willing” to the question “Based on what we have described, how willing would you be to participate in this study?” the response was considered to be “Willing to participate.”

^b^N/A: not applicable.

### Willingness to Participate in the Proposed Study

In the survey, respondents were asked about their willingness to participate in the study overall and in specific aspects of the proposed study. Most respondents (67/82, 82%) indicated that they would be willing to participate in the study based on the description in the survey: 37% (30/82) indicated that they were very willing, and 44% (36/82) indicated that they were somewhat willing (Table 2). When asked about specific aspects of the study, willingness to participate varied. Of the 67 respondents willing to participate in the study, 62 (93%) indicated that they were willing to join an Instagram group moderated by a health coach to help better control asthma. Slightly fewer respondents (49/67, 73%) were also willing to post about their condition on an account created for the purpose of the study and interact with other participants’ posts (61/67, 91%). The aspect of the study most popular among participants (64/67, 96%) was the proposal to write a short reflection about their asthma experience every month for 3 months. Table 2 further describes the willingness of survey respondents to participate in the proposed study.

### Concerns and Interests

Few respondents (15/82, 18%) indicated that they had no concerns about participating in the proposed study. The most common concern reported was “I don’t want to share photos relating to my asthma with others” (37/82, 45%). Respondents (28/82, 34%) also indicated that they needed more information. Some respondents believed that they did not have the time to participate (15/82, 18%), that the study would not help them (11/82, 13%), or that compensation was inadequate (12/82, 15%).

Survey respondents also provided comments about aspects of the study that concerned and interested them. The written comments had 4 underlying themes: utility, social support, compensation, and privacy (Table 3). The utility of the study was highlighted by multiple comments expressing interest in the possibility of asthma improvement as well as openness to new methods of asthma control. Some respondents also hoped that the study would help other individuals with asthma and add to research in the field. With respect to social support, respondents were interested in the idea of connecting with other individuals with asthma on Instagram. They expressed interest in both providing and receiving social support related to asthma management. One respondent said they would “Just like to chat with others who have asthma.” Respondents generally expressed interest in receiving some form of compensation for participating in the proposed study. The primary concern was privacy; some respondents expressed concern about sharing their asthma experience with other individuals who had asthma.

**Table 3:** Emergent themes from survey comments.

Theme	Illustrative quote
Utility	I think it would be interesting to see what could possibly help me manage my asthma better. Due to my current situation I am always interested in new possibilities. I’m most interested in using social media as a tool to help people with asthma.
Social support	I think it’s interesting to develop a discussion around asthma on Instagram, specifically. Might benefit a lot of people to talk about their experiences.
Compensation	Earning money. The compensation.
Privacy	I don’t want my identity shared publicly.

## Discussion

### Principal Findings

The results of our survey suggest that among young adult Instagram users with poorly controlled asthma, there is substantial interest in participating in a study that will use Instagram to connect participants with peers and a health coach to share information about self-management of asthma and build social connection. The majority of respondents (67/82, 82%) indicated that they would be willing to participate in such a pilot study on Instagram. Social media has helped create community without propinquity. Researchers have used this medium to deliver peer support and evidence-based programs through novel channels such as Twitter, Instagram, Facebook, and study-specific apps and websites [7-9,13]. For example, Facebook groups have been used to promote healthy gestational weight gain during pregnancy. A pilot study recruited 19 postpartum participants to receive a 12-week intervention based on the Diabetes Prevention Program via a private Facebook group. Results were promising in that clinically significant weight loss was documented in 58% of participants and 82% were likely to recommend the program to a friend [14]. The results of our survey are consistent with the findings of this study in that many adults are interested in receiving health care interventions through social media.

The proposed study would meld the sharing of evidence-based knowledge on self-management of asthma with the principles of photovoice and digital storytelling. Both photovoice and digital storytelling are grounded in community-based participatory research with the motivating goal of empowering participants to advocate for change in their communities [15,16]. Both interventions are traditionally delivered in an in-person group setting where participants provide real-time support to one another. There is evidence that digital storytelling may benefit individual storytellers by improving social support, self-efficacy, and emotional acceptance, which in turn encourage healthy behaviors [17]. Our proposed intervention deviates from these traditional models in its medium of delivery (ie, through a social media platform), but shares the conceptual foundation that sharing of images and engaging in storytelling and self-reflection may empower participants to make positive changes both in their lives and in the larger community. A pilot study, similar to the one proposed in this survey, explored the feasibility of using in-person focus groups and Instagram to adapt the principles of photovoice to social media for adolescents with type 1 diabetes and examined the types of photos shared in this setting [12]. The study, however, did not examine health outcomes. Our proposed pilot trial would aim to test whether an Instagram-delivered intervention based on the principles of photovoice and digital storytelling would improve clinical outcomes in participants with asthma.

An unexpected finding of the survey was that respondents were most enthusiastic about the written reflection proposed as part of the study. This suggests the potential for an online community to move beyond simply posting about their asthma experience toward engaging in reflection and dialogue, which are the core aspects of photovoice and digital storytelling. The study as proposed does not include any in-person interaction; thus, a challenge will be creating a community where individuals feel comfortable sharing with their peers and the research team.

Understanding respondents’ answers regarding Instagram use as well as their interests and concerns surrounding the study design will aid the implementation of our proposed pilot study. Privacy was a common concern among respondents: approximately half (46/82, 56%) used private Instagram accounts and some (37/82, 45%) indicated that they did not want to share photos related to their asthma experience publicly. To accommodate these concerns, study participants could create new private Instagram accounts for the purpose of the study. Participants would only follow other study participants and the health coach, thus creating a “private group,” which would prevent study-related posts from appearing on their existing Instagram accounts.

### Limitations

Our study has several limitations. It used a convenience sample based on an advertisement placed on Instagram, so our findings may not be generalizable to the larger population of individuals with asthma on other social media apps. Most of our respondents were young adults with high levels of education, which reflects the demographics of Instagram users. However, because the proposed study would be completely administered through Instagram, the survey sample only reflects those most likely to participate in the study. It may be worth exploring potential online interventions that use more ubiquitous social media apps such as Facebook to reach older adults with asthma.

### Conclusion

The findings of this study indicate substantial interest among young adult Instagram users with poorly controlled asthma to participate in a pilot randomized controlled trial that will use Instagram to deliver social support and information on self-management of asthma. The respondents’ expressed interests and concerns should guide the implementation of this pilot as well as that of similar future trials. Considering the prevalence of asthma and the increasing reliance on social media for health information, it is important to understand how health care providers can use platforms such as Instagram to improve self-management of asthma.

## Appendix

Multimedia Appendix 1 Facebook recruitment advertisements.

Multimedia Appendix 2 Study survey given to participants.
