# Supplementary material for: Evaluation of the Acceptability of a Proposed, Instagram-Based, Randomized Controlled Trial for People With Asthma: Survey Study
Source: JMIR Form Res. 2021 Sep 30;5(9):e24005. doi: 10.2196/24005 (PMC8517815; doi:10.2196/24005)
Supplement: Multimedia Appendix 1 [file formative_v5i9e24005_app1.pdf]

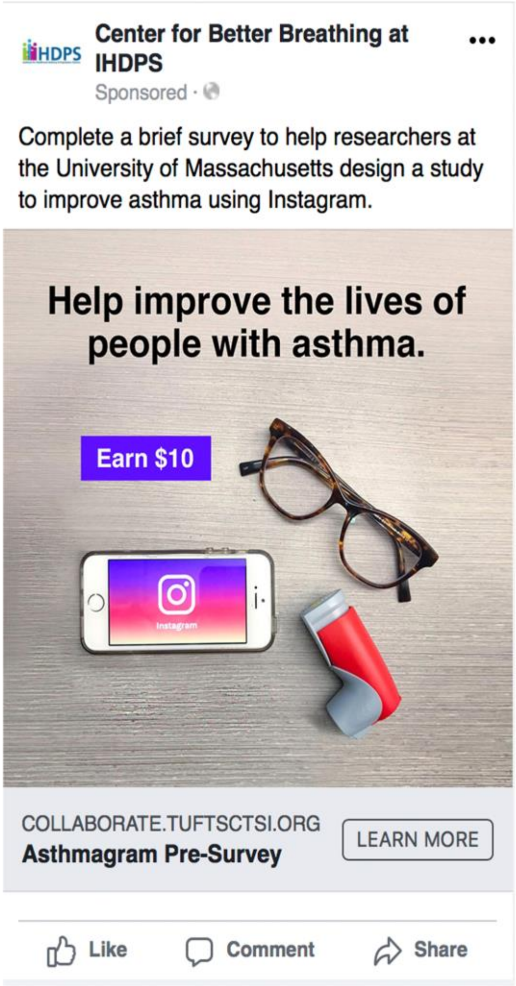
IHDPS
Center for Better Breathing at IHDPS
Sponsored ·
Complete a brief survey to help researchers at the University of Massachusetts design a study to improve asthma using Instagram.
Help improve the lives of people with asthma.
Earn $10
Instagram
COLLABORATE.TUFTSCTSI.ORG
Asthmagram Pre-Survey
LEARN MORE
Like
Comment
Share

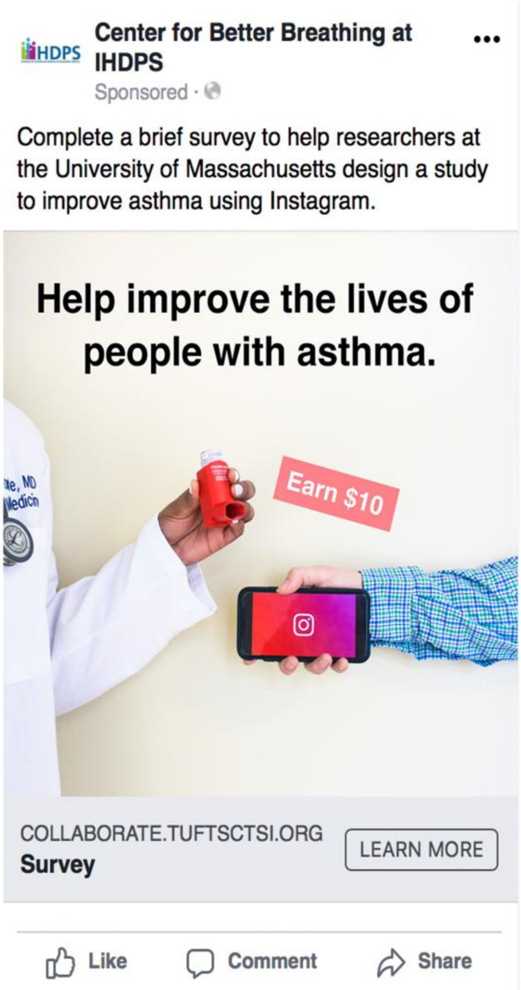
IHDPS
Center for Better Breathing at IHDPS
Sponsored ·
Complete a brief survey to help researchers at the University of Massachusetts design a study to improve asthma using Instagram.
Help improve the lives of people with asthma.
Earn $10
COLLABORATE.TUFTSCTSI.ORG
Survey
LEARN MORE
Like
Comment
Share
