# Supplementary material for: Evaluation of the Acceptability of a Proposed, Instagram-Based, Randomized Controlled Trial for People With Asthma: Survey Study
Source: JMIR Form Res. 2021 Sep 30;5(9):e24005. doi: 10.2196/24005 (PMC8517815; doi:10.2196/24005)
Supplement: Multimedia Appendix 2 [file formative_v5i9e24005_app2.pdf]

Asthmagram Survey

Based on your initial resposnes, you are eligible to complete our survey. Please read the agreement below.

Thank you!

You are being asked to participate in a brief survey for research purposes. The goal of this research study is to understand the willingness of Instagram users living with asthma to participate in an intervention that requires participants to share about their asthma online and interact with a health coach and other Instagram users with asthma. We expect to survey approximately 100 people. The survey will take about 10 minutes of your time. Your participation in this study is voluntary and your decision will have no impact on your relationship with the Institute for Healthcare Delivery and Population Science (IHDPS) Center for Better Breathing at the University of Massachusetts Medical School - Baystate.

Completing the survey will earn you a 1-in-10 chance to win a $10 Amazon gift card. If you win, you will receive an email containing the gift card within 3 business days.

The survey includes some demographic questions that may be sensitive or personal. You are free to select "Prefer not to answer" on these questions for any reason. There is a minimal risk in participating in this survey but there is always the risk of loss of confidentiality. Your survey responses will be recorded and stored on REDCap. Only members of the research team will be able to access this information. You may not benefit from this study, but the information gathered from this survey may help in implementing a future study that aims to improve asthma control through social media.

By agreeing to continue with this survey, you are consenting to participate in this research study with the understanding that you are free to withdraw at any time. If at any time you wish to discontinue your participation, you may exit the survey and it will not result in any penalty.

If you have any questions about this study, please contact Brent Heineman at 413-794-6606.

If you would like to discuss your rights as a research participant, or wish to speak with someone not directly involved in this study, please contact Baystate Medical Center Institutional Review Board at (413) 794-4356.

Version # 1.0
Date: 11/1/2019

Please read the following:

I have read and understand this study agreement:

- ◯ Yes
- ◯ No

Questions about your asthma:

In the last year, have your visited the emergency department for the purpose of treating your asthma?
- ◯ Yes
- ◯ No

In the last year, have you been hospitalized for your asthma?
- ◯ Yes
- ◯ No

	I am not at all confident	I am a little confident	I am somewhat confident	I am quite confident	I am very confident
I can manage my symptoms during my daily activities.	◯	◯	◯	◯	◯
I can keep my symptoms from interfering with relationships with friends and family.	◯	◯	◯	◯	◯
I can manage my symptoms in a public place.	◯	◯	◯	◯	◯
I can work with my doctor to manage my symptoms.	◯	◯	◯	◯	◯

**Questions about your Instagram use:**	
How frequently do you open the Instagram app/log-onto Instagram?	◯ Multiple times a day<br>◯ About once a day<br>◯ A few times per week<br>◯ A few times per month
Do you post on Instagram?	◯ Yes<br>◯ No
If yes, how frequently?	◯ Daily<br>◯ Weekly<br>◯ Monthly<br>◯ Yearly
Do you comment on posts?	◯ Yes<br>◯ No
If yes, how frequently?	◯ Daily<br>◯ Weekly<br>◯ Monthly<br>◯ Yearly
Do you have a public or private Instagram account?	◯ Public<br>◯ Private
Do you follow any hashtags or accounts related to Asthma?	◯ Yes<br>◯ No
Do you follow any hashtags related to health and wellness?	◯ Yes<br>◯ No

Questions about a future research study:

We are planning a study to determine whether people with asthma benefit from sharing and reflecting on their condition using social media tools like Instagram. As part of this intervention we will ask participants to follow a health coach from the study team on Instagram. The coach will post regularly about asthma control and management. These posts will prompt participants to discuss living with asthma with other participants. The study will last 3 months. At the end of each month, participants will complete a self-reflection exercise about their asthma and experiences in the study.

Participants will be compensated $75 for completing the study.

As part of the study, how willing would you be to join an Instagram group, moderated by a health coach, with the goal of helping participants better control their asthma?

- ◯ Very Willing
- ◯ Somewhat Willing
- ◯ Somewhat Unwilling
- ◯ Very Unwilling

How willing would you be to post about your asthma on an account created for the purposes of the study?

- ◯ Very Willing
- ◯ Somewhat Willing
- ◯ Somewhat Unwilling
- ◯ Very Unwilling

How willing would you be to like, comment, or interact with the posts of others with asthma on a weekly basis?

- ◯ Very Willing
- ◯ Somewhat Willing
- ◯ Somewhat Unwilling
- ◯ Very Unwilling

How willing would you be to write a short paragraph reflecting on your experiences with asthma once per month for three months?
These reflections would only be shared with the research team.

- ◯ Very Willing
- ◯ Somewhat Willing
- ◯ Somewhat Unwilling
- ◯ Very Unwilling

Based on what we have described, how willing would you be to participate in this study?

- ◯ Very Willing
- ◯ Somewhat Willing
- ◯ Somewhat Unwilling
- ◯ Very Unwilling

What are your concerns about participating in the study described above, if any?

- ☐ I don't want to share photos relating to my asthma with others.
- ☐ I don't think the compensation is enough.
- ☐ I don't use Instagram enough.
- ☐ I don't think this study will help me.
- ☐ I don't have the time.
- ☐ I need more information.
- ☐ Other
- ☐ No concerns

Other, please specify: ______________________________

Please comment on what (if any) elements of the proposed study interest you. ______________________________

Please use the provided space to share any additional thoughts you may have about how to make the proposed study better. ______________________________

May we contact you within the next 12 months to invite you to participate in the study?

- ◯ Yes
- ◯ No

Questions about your demographics:

What is the highest degree or level of school you have completed?	◯ Less than high school degree<br>◯ High school degree or equivalent (e.g. GED)<br>◯ Some college but no degree<br>◯ Associate degreee<br>◯ Bachelor degree<br>◯ Graduate degree<br>◯ Prefer not to answer
Current gender identity:<br>How do you describe yourself?	◯ Male<br>◯ Female<br>◯ Transgender<br>◯ Do not identify as male, female, or transgender<br>◯ Prefer not to answer
Ethnicity:	◯ Not Hispanic/Latino<br>◯ Hispanic/Latino<br>◯ Prefer not to answer
Race:	◯ American Indian or Alaska Native<br>◯ Asian<br>◯ Black or African-American<br>◯ Native Hawaiian or Other Pacific Islander<br>◯ White<br>◯ Other (please specify)<br>◯ Prefer not to answer
Other (please specify):	__________________________________

End of survey

Thank you for completing this survey. If you would like a chance to win a $10 Amazon gift card, please provide your email address.
If you win, you will be sent an email containing the gift card within 3 business days.

____
